# Particularities of Offenders Imprisoned for Domestic Violence from Social and Psychiatric Medical–Legal Perspectives

**DOI:** 10.3390/medicina57111187

**Published:** 2021-11-01

**Authors:** Oana-Maria Isailă, Sorin Hostiuc, Mihai Teodor, George-Cristian Curcă

**Affiliations:** 1 Department of Legal Medicine and Bioethics, Faculty of Dental Medicine, “Carol Davila University” of Medicine and Pharmacy, RO-020021 Bucharest, Romania; soraer@gmail.com; 2 “Mina Minovici” National Institute of Legal Medicine, RO-042122 Bucharest, Romania; mihai.teodor@inml-mm.ro (M.T.); cgcurca@yahoo.com (G.-C.C.); 3 Department of Legal Medicine and Bioethics, Faculty of Medicine, “Carol Davila” University of Medicine and Pharmacy, RO-020021 Bucharest, Romania

**Keywords:** psychiatric medical–legal reports, interpersonal violence, domestic violence, imprisoned perpetrators, psychiatric diagnosis

## Abstract

*Background and Objectives:* It is known that there may be an interconditionality between social status, personality disorders, and aggressive behavior. This study aimed to analyze the social and psychiatric diagnosis characteristics in subjects imprisoned for domestic violence acts compared to other types of aggressive behaviors. *Materials and Methods:* We performed a retrospective study using psychiatric medical–legal reports at the National Institute of Legal Medicine “Mina Minovici” Bucharest from 2016 to 2020. *Results:* We included 234 cases in our analysis, from which 132 (56%) were domestic violence offenders (DVO), and 102 (44%) were violence offenders imprisoned for other aggressions (OVO). Overall, DVOs were older than OVOs (43.0 +/− 14.7 vs. 36.1 +/− 16.6 years-old). In both study groups, most subjects were men, but the DVO group had more women than the OVO group: 23 cases (17%) and 3 cases (3%), respectively. In 14 cases (11%), previous criminal records were found from the DVO and 31 (30%) from the OVO group. Significantly fewer DVO were chronic psychoactive substance users: 83 (63%) in the DVO group versus 78 (86%) in the OVO group. Significantly more DVO had suicidal tendencies 26 (20%) compared to OVO 9 (9%). DVO subjects had significantly less often unsocialized conduct disorder or antisocial personality disorder compared to the OVO group. *Conclusions:* We found that DVO, compared to the OVO, were more numerous, older, less abusive, with a less frequent history of psychoactive substance abuse and addictions, and were less frequently indifferent to the committed acts.

## 1. Introduction

The prevalence of personality disorders around the world is approximately 7.8% and is correlated with the national development index [1]. Moreover, previous studies have revealed a direct correlation between aggressive behavior and personality disorders [2,3,4] or socio-economic levels [5,6].

Aggressive behavior can be the basis of interpersonal violence and implicitly of domestic violence. Domestic violence refers to physical, psychological, verbal, sexual, economic, social, spiritual, and cyber aggression of a family member [7]. Among the aforementioned forms of domestic violence, physical violence is medical–legal objectifiable by examining the victim. In Romania, preexisting medical–legal studies on domestic violence analyzed the victims and revealed the predominance of women victims, with traumatic-injury topography on the head [8] and upper limbs [9].

On a national level, there are no similar recent studies about domestic violence offenders (DVOs) from a medical–legal perspective. The last statistics available from the Romanian Police (2019) reported 26.239 perpetrators who had caused injuries and other types of domestic violence, of whom 91% were men and also the victim’s partner in the majority of cases. Further, 82 perpetrators had committed homicide, of whom 75.5% were men and a distant relative of the victim in the majority of cases [10]. Further, the study conducted in 2012 by Stoleru et al. found that less than 20% of domestic violence criminal cases resulted in a court case, of which 62.16% resulted in a conviction, and 16.22% of cases resulted in custodial sentences [11].

The aim of this study was to analyze the socially and psychiatrically diagnosed characteristics of subjects imprisoned for acts of domestic violence compared to other types of aggressive behaviors.

## 2. Materials and Methods

We performed a retrospective study using psychiatric medical–legal reports submitted to the National Institute of Legal Medicine “Mina Minovici” Bucharest between 2016 and 2020. The inclusion criteria were as follows: prisoners condemned for acts of fatal and non-fatal physical aggression. Reports not analyzing these aspects were excluded from the study. Detainees who met the inclusion criteria were divided in two groups: group 1 domestic violence offenders (DVOs) where the victim was a family member, such as a blood relative, spouse, or long-term partner and group 2 (other violence offenders (OVOs) in other contexts of interpersonal violence, such as when the victim is unknown to the aggressor, a colleague, neighbor, or friend). Details about the circumstances, type of aggression, and victim were obtained from police reports and/or personal histories obtained during medical-legal examinations.

Each offender was subjected to an exhaustive examination performed by a committee, with different specialists periodically, composed of a legal medicine physician and two psychiatrists with experience in forensic psychiatry. A psychological examination was also performed. These examinations are usually requested to establish the presence/absence of competence and are conducted after offenders have been hospitalized in psychiatric penitentiary hospitals for preliminary evaluations.

The most frequently used tests for the psychological evaluations were the Woodworth–Mathews psychoneurotic tendency questionnaireWoodworth-Mathews psychoneurotic tendency questionnaire, Structured Clinical Interview for DSM Disorders (SCID), and projective tests for personality measurement (drawing, Szondi, and Rorschach). The following variables were analyzed: age and gender of the offender, relationship between the offender and the victim, presence of multiple victims, criminal record, level of education, profession, marital status, history of psychiatric and non-psychiatric diseases, substance abuse or suicidal tendencies, perpetrator’s attitude toward the act they have committed, the perpetrator’s intellect, intellectual efficiency, psychiatric diagnosis, criminal competence, and the need for safety measures to diminish the social danger posed by the offender.

The study was approved by the institutional ethics board, and the IRB outcome code is 972/26.01.2021.

Data extracted from the medical–legal psychiatric reports were systematized using Excel 2007, and the resulting database was processed with Jamovi v 1.2.27. We used ANOVA to detect significant differences between groups and the chi-square test (X^2^) to analyze associations between categorical variables.

## 3. Results

### 3.1. Socio-Demographic Aspects

Of the 234 offenders included in the study, 132 (56.4%) were imprisoned as a result of an act of domestic violence (DVO), and 102 (43.6%) were imprisoned as a result of another type of interpersonal violence (OVO). The average age of the subjects was 40.0 +/− 15.9 years. Of these, the DVOs had an average age of 43.0 +/− 14.7 years, which was significantly higher than the OVOs who had an average age of 36.1 +/− 16.6 years (ANOVA, F = 11.5, *p* < 0.001).

Most of the subjects were male, with 83% (109 cases) in the DVO group and 97% (99 cases) in the OVO group. The association between the perpetrator’s gender and the type of aggression was statistically highly significant (chi-square = 12.2, *p* < 0.001). Moreover, the odds ratio (OR) for a woman to be the aggressor in domestic violence cases (rather than other types of hetero-aggression) was 6.96, with a CI (95%) of 2.03–23.9.

In the DVO group, 66 (50%) originated from a rural environment, and 66 (50%) were from an urban environment. In the OVO group, 44 (43%) originated from a rural environment, and 58 (58%) were from an urban environment. Accordingly, the association between the aggressors’ environment of origin and the type of aggression context was not statistically significant.

With reference to the degree of education of the inmates in the study groups, 19 (14%) had a higher education, 26 (20%) attended trade school, 21(16%) had finished high school, 34 (26%) finished eight years of study (primary school), and 32 (24%) had no formal education in the DVO group. By contrast, 7 (7%) had a higher education, 12 (12%) attended trade school, 20 (20%) finished high school, 27 (26%) finished eight years of study (primary school), and 36 (35%) had no formal education in the OVO group. Although there were some differences, the association between the degree of education of the offender and the context of aggression was not statistically significant, with people having a medium or higher education being poorly represented in both groups.

At the time of committing their act, 69% (91) from the DVO group were unemployed, 22% (28) were employed, and 9% (12) were retired. By comparison, 77% (79) were unemployed, 12% (12) were in employment, and 11% (11) were retired. The association between professional status and type of aggression was not statistically significant, and unemployed people were predominant in both groups.

With regard to marital status, 75 (57%) were not married, 33 (25%) were married, 17 (13%) were divorced, and 7 (5%) were widowers in the DVO group, while 70 (69%) were unmarried, 17 (17%) were married, 10 (10%) were divorced, and 5 (5%) were widowers in the OVO group. The association between the perpetrator’s marital status and aggression type was not statistically significant.

### 3.2. Aspects in Relation to the Aggression

With respect to the number of victims within the same act of aggression, 91% (120) of cases in the DVO group involved only one victim, and 9% (12) involved multiple victims. By comparison, 82% (84) of cases involved one victim, and 18% (18) involved multiple victims in the OVO group. This difference was not statistically significant.

### 3.3. The Criminal Records of the Perpetrators

Of the inmates in the DVO group, 14 (11%) had criminal records, and 118 (89%) had no previous convictions compared to 31 (30%) and 71 (70%), respectively, for the OVO group. This difference was highly statistically significant (chi-square = 14.5, *p* < 0.001). The odds ratio OR that a DVO with a criminal record was an aggressor in domestic violence (and not in other hetero-aggressions) was 3.68 lower compared to OVO cases with a CI (95%) of between 1.83 and 7.38.

There were 83 (63%) chronic substance abusers in the DVO group compared to 78 (76%) in the OVO group with a statistically significant difference (chi-square) of *p* = 0.026. The number of chronic ethanol abusers was 72 (55%) in the DVO group and 71 (70%) in the OVO group with a statistically significant difference (chi-square) of *p* = 0.019. Moreover, 9 (9%) of offenders in the OVO group were chronic abusers of ethanol combined with other drugs with a statistically significant difference (chi-square) of *p* < 0.001. Other types of drugs of abuse encountered in our study included the following: legal highs (6 (5%) DVOs, 3 (3%) OVOs); cannabis (4 (3%) DVOs, 7 (7%) OVOs); volatile substances (0 (0%) DVOs, 4 (4%) OVOs); cocaine (2 (2%) DVOs, 3 (3%) OVOs); methadone (1 (1%) in both groups); heroin (3 (2%) DVOs, 2 (2%) OVOs; LSD (1 (1%) DVO, 2 (2%) OVOs). The only statistically significant difference (chi-square) was in the case of volatile substances (*p* = 0.022). At the moment of aggressive behavior leading to imprisonment, 26 (20%) DVOs and 22 (22%) OVOs had behavioral disorders due to alcohol consumption; 4 (3%) DVOs and 5 (5%) OVOs exhibited behavioral disorders due to drug consumption; and 19 (14%) DVOs and 16 (16%) OVOs had an addiction syndrome. The differences were not statistically significant.

There were no statistically significant differences between the two groups pertaining to the inmates’ pathological and psychiatric backgrounds.

### 3.4. Attitude towards the Act

In the DVO group, autolytic ideation and suicide attempts were observed in 26 (20%) cases and absent in 106 (80%). By contrast, suicidal ideation was present in 9 (9%) cases and absent in 93 (91%) in the OVO group, which was a statistically significant difference (chi-square = 5.35, *p* = 0.021). The OR was 2.53 with the CI (95%) between 1.13 and 5.68.

With regard to the committed act, 72 (55%) of the DVO group stated that they regretted the act, 42 (32%) were indifferent, 12 (9%) made no comment, 2 (1%) stated that they did not remember committing the act, and 4 (3%) denied committing the act. By comparison, 44 (43%) regretted the act, 44 (43%) were indifferent, 4 (4%) made no comment, 9 (9%) denied committing the act, and 1 (1%) refused psychiatric and psychological examinations in the OVO group. The association between attitude towards the act and the type of aggression was statistically significant (chi-square = 12.1, *p* = 0.034. df = 5).

### 3.5. Characteristics of the Perpetrator

With regard to intellectual efficiency, 77 (58%) DVOs had an intellect corresponding to their level of education and environmental background, 4 (3%) were under their level of education, 4 (3%) had mental retardation, 47 (36%) had low intellect, and no-one had medium intellect. By contrast, 54 (53%) OVOs had an intellect corresponding to their level of education and environmental background, 4 (4%) were under their level of education, 1 (1%) had mental retardation, 41(40%) had low intellect, and 2 (2%) had medium intellect. The association between the perpetrator’s intellectual efficiency and the type of aggression did not exhibit any statistically significant difference.

In the DVO group, 120 (91%) did not simulate their behavior, while 12 (9%) had simulated delirious ideation. In the OVO group, 89 (87%) did not simulate their behavior, and 13 (13%) had simulated delirious ideation. This association did not exhibit any statistically significant difference.

### 3.6. Psychiatric Diagnosis

Of the DVOs, 3 (2%) had unsocialized conduct disorder, while 10 (10%) of the OVOs had the same diagnosis with a statistically significant difference (chi-square = 6.22, *p* = 0.013). Further, the OR that a person who assaults a person from their family would have unsocialized conduct disorder was 4.67 lower, with a CI (95%) of 1.25–17.5.

An antisocial personality disorder diagnosis was encountered in 17 cases (13%) in the DVO group, compared with 33 cases (32%) in the OVO group, which was statistically highly significant (chi-square = 13.0, *p* < 0.001). The OR that a person who assaults someone from their family has an antisocial personality disorder was 3.24 lower, with a CI (95%) of 1.68–6.24.

In the present study there were no statistically significant differences in the cases of the other psychiatric diagnoses between the DVO and OVO groups. Further, there were no statistically significant differences in what concerns enforcing safety measures.

Competence was maintained in 103 (78%) of the DVOs, was low in 13 (10%), and absent in 16 (12%) compared to 88 (86%), 10 (10%), and 4 (4%) for the OVOs, respectively. This difference was not statistically significant.

## 4. Discussion

In this study, we comparatively analyzed DVO and OVO imprisoned subjects through socio-demographic aspects and psychiatric diagnostic elements of this group. We found a greater number of aggressors in the context of domestic violence in the DVO group (132 (56.4%)) compared to the OVO group (102 (43.6%)). Given that interpersonal violence is usually perceived as an inevitable phenomenon throughout life on both individual and societal levels [12], it is more frequently found within the family unit. This is because the victim is in close proximity, the aggressor has a stronger desire to be in control in this familiar setting [13], and because of the victim’s higher level of tolerance [14].

The average age of the subjects included in the study was 40.0 +/− 15.9, although offenders in the context of domestic violence had a significantly higher average age than offenders in other types of hetero-aggression (43.0 +/− 14.7 years compared to 36.1 +/− 16.6 years). In a previous study conducted in Germany by C. Graz et al., which was based on the national criminality registry of aggressors with prior hospitalizations for affective disorders, the resultant median age was 53.26 +/− 16.3 years [15]. This was above the average age of the participants in this study. Further, within a study that specifically targeted male aggressors in the context of domestic violence by C. Peek-Asa et al., 35–44 years of age was established as the age of abuse [16]. This result includes the average age in our study. In a similar study, Rode et al. focused exclusively on male and female perpetrators in cases of domestic violence. They found the age of abuse to be 36.92 +/− 10.73 years [17], which also coincides with the results of the present study. Moreover, Shorey et al. established that the average age of males arrested for domestic violence is 33.1 +/− 10.1 years [18].

While most subjects were men in both study groups, there were more women in the DVO group compared to the OVO group (23 (17%) compared to 3 (3%)). Previous studies that have analyzed the perpetrators in cases of interpersonal violence also discovered that the majority of aggressors were male [15,17,19,20]. Again, these results are consistent with the current study. The higher number of female DVOs compared to female OVOs can be partly explained by the existence of infanticide cases (the present study contained five such cases). In the other cases, the victim was the intimate partner, and the aggression was a result of the female partner trying to defend herself, or the aggression was sustained by repeated psychological abuse. While some specialized literature mentions female aggressors in the context of self-defense [21,22], other studies have exposed females as aggressors with an offensive purpose [23,24].

There were no statistically significant differences between origin, degree of education, professional insertion, and marital status. In both groups, most of the perpetrators had a low level of education, were unemployed, and were not legally or officially married. These results correspond with conclusions of other studies in which aggressive behavior was caused by social marginalization and low socio-economic status—factors that can generate tense and stressful situations [5,25,26,27]. The study of Hing et al. highlighted the financial abuse in domestic violence, an aspect that increases the socio-economic difficulties of the family environment [28]. In connection with and as a consequence of these social aspects, the intellectual efficiency of the subjects in both study groups (and the majority of cases) corresponded to their level of education and environment of origin, without statistically significant differences.

Although there were fewer cases with multiple victims (two or more victims in the same act of aggression) in the DVO group (12 (9%)) compared to the OVO group (18 (18%)), this difference was not statistically significant. In a previous study, J. Abolarin et al. concluded that multiple homicides carried out at the same time targeted relatives of the aggressor [29].

Previous criminal records were found in 14 (11%) DVOs and 31 (30%) OVOs, which is a statistically significant difference and suggests that the risk of re-offending is lower for DVOs. In previous studies concerning the relationship between criminal records and aggression on an intimate partner, Piquero et al. revealed an association between these two elements [30], and Bauto et al. found in their study that most of domestic violence perpetrators (65.8%) had previous criminal records [31]. This result was different to the one in this present study, which is probable because many cases of domestic violence remain unreported [8].

While there were chronic psychoactive substance abusers in both study groups, there were fewer in the DVO group—a difference that was statistically significant. In the present study, OVOs were exclusively abusers of volatile substances and alcohol combined with other drugs. Further, few previous studies have presented comparative analyses in this regard. In a similar study where substance abuse in aggressors who assaulted their intimate partner were compared to aggressors in cases of violence, Kranen et al. revealed the predominance of psychoactive substance abuse in cases of perpetrators of general violence (61.5% compared to 30.9%) [32].

Autolytic ideation, attempted suicide, and regret of committing the act were present in the majority of aggressors in DVOs, and the difference was statistically significant. Moreover, previous studies on murder–suicide cases have indicated suicide attempts in cases of aggressors in a domestic context [33]. Autolytic ideation is based on intrinsic, individual factors in addition to external, contextual ones. The study carried out by Fridel and Zimmerman indicated “(1) that living in a disadvantaged place and state decreased the likelihood of suicide after a homicide, and (2) that the ability of the victim-offender relationship to distinguish homicide only and homicide-suicide was marginalized in disadvantaged areas” [34].

Generally, according to Bauto et al., there is an interconditionality between neuroticism and aggressive behavior in the case of domestic violence suspects [31], and neuroticism is a predictor of many psychiatric pathologies [35]. With regard to psychiatric diagnoses in the cases of inmates who are incarcerated on homicide charges, previous studies have indicated an increased prevalence of schizophrenia [36,37,38], depression, personality disorders [37,39,40], affective disorders, and organic personality disorders [2]. Moreover, in a study concerning psychiatric diagnoses in cases of domestic violence, Yu et al. determined that all psychiatric pathologies (with the exception of autism) have been associated with an increased risk of domestic violence. Of all these pathologies, depressive disorders, anxiety, disorders due to alcohol abuse, attention deficit hyperactivity disorder (ADHD), and personality disorders were more frequently associated with domestic violence [41]. Further, Garcia-Jimenez et al. concluded in a similar study that domestic abusers more frequently had personality disorders such as borderline disorders or antisocial personality disorders [5]. In a study concerning the prevalence of psychiatric problems in men arrested for domestic violence, Shorey et al. obtained high numbers for the following diagnoses: posttraumatic stress disorder (PTSD), depression, generalized anxiety disorder, and personality disorders due to the consumption of drugs and/or alcohol [18]. Our study has revealed significant differences between DVOs and OVOs for the unsocialized conduct disorder diagnosis, being less frequently encountered in DVO: 2 (2%) compared to 10 (10%). This diagnosis was formulated in the case of perpetrators aged under 20 years; there were fewer subjects from this age group in the DVO group. The antisocial personality disorder diagnosis was less frequent in the DVO group (17 (13%) compared to 33 (32%) in the OVO group), which was a statistically significant difference. The unsocialized conduct disorder (in the case of minors who have committed criminal acts) turns in most cases in adulthood into antisocial personality disorder.

Competence is the mental capacity of a person to understand the meaning of their actions and their consequences. This did not reveal any statistically significant differences between the DVO and OVO groups, even though it was absent in more DVOs (16 (12%) compared to 4 (4%)).

### 4.1. Limits of the Study

This study’s limits were represented by the small number of cases, by the inequality in the number of cases in the study groups, and by the absence of a social inquiry in some cases.

### 4.2. Applicability

The results of the present study suggest a need to reach and exceed the subsistence level and to facilitate access to specialized medical examinations, psychological counseling, and psychiatric consultation, to prevent the phenomenon of domestic violence and of interpersonal violence in general. Further, collaboration between institutions is of vital importance to create plans for intervention centered on access to education, social insertion, and prevention of substance abuse in the disadvantaged population.

## 5. Conclusions

A linked interconditionality exists between low socio-economic status, low level of education, personality disorders, inadequate social insertion, and aggressive behavior. These elements were found in both study groups. Compared to OVOs, DVOs were more numerous, older, more frequently had autolytic ideation and regretted committing the act, had significantly less often unsocialized conduct disorder or antisocial personality disorder, were less abusive (having a lower number of criminal records), and had less frequent histories of psychoactive substance abuse and addiction.

## Data Availability

The datasets used and/or analysed during the current study are available from the corresponding author on reasonable request.

## References

[1] Winsper C., Bilgin A., Thompson A., Marwaha S., Chanen A.M., Singh S.P., Wang A., Furtado V. (2020). The prevalence of personality disorders in the community: A global systematic review and meta-analysis. Braz. J. Psychiatry.

[2] Valença A.M., De Moraes T.M. (2006). Relationship between homicide and mental disorders. Braz. J. Psychiatry.

[3] Hodgins S., Mednick S.A., Brennan P.A., Schulsinger F., Engberg M. (1996). Mental disorder and crime. Evidence from a Danish birth cohort. Arch. Gen. Psychiatry.

[4] Swanson J.W., Holzer C.E., Ganju V.K., Jono R.T. (1990). Violence and psychiatric disorder in the community: Evidence from the Epidemiologic Catchment Area surveys. Hosp. Community Psychiatry.

[5] García-Jiménez J.J., Godoy-Fernández C., Llor-Esteban B., Ruiz-Hernández J.A. (2014). Differential profile in partner aggressors: Prison vs. mandatory community intervention programs. Eur. J. Psychol. Appl. Leg. Context.

[6] Fulu E., Jewkes R., Roselli T., Garcia-Moreno C. (2013). Prevalence of and factors associated with male perpetration of intimate partner violence: Findings from the UN multi-country cross-sectional study on men and violence in Asia and the Pacific. Lancet Glob. Health.

[7] Law no. 217/2003 on the Preventing and Fighting against Domestic Violence (Officially Published in MO, Part I, no 367/2003 in Force from 27 August 2003). http://legislatie.just.ro/Public/DetaliiDocument/44014.

[8] Curca G.C., Dermengiu D., Hostiuc S. (2012). Patterns of Injuries in Domestic Violence in a Romanian Population. J. Interpers. Violence.

[9] Isaila O.-M., Hostiuc S., Curca G.-C. (2021). Relationship between Injury Pattern and Domestic Violence in a Romanian Population. Am. J. Forensic Med. Pathol..

[10] Romanian Police (2020). Domestic Violence Statistics 2019. https://violentaimpotrivafemeilor.ro/statistici-violenta-in-familie-2019/.

[11] Stoleru M., Antal I., Dávid-Kacsó Á. (2012). The Approach of Domestic Violence by the Criminal Justice System in Romania. Rev. Asistenţă Soc..

[12] Sethi D., Butchart A., Heggenhougen H.K. (2008). Violence/Intentional Injuries—Prevention and Control. International Encyclopedia of Public Health.

[13] Curcǎ G.C., Buda O., Cǎpǎţânǎ C., Marinescu M., Hostiuc S., Dermengiu D., Cârţînǎ C., Creţoiu V.A., Stoica N.A., Bǎdescu I. (2008). Study on domestic violence: A legal medicine perspective. Rom. J. Leg. Med..

[14] Gracia E., Herrero J. (2006). Acceptability of domestic violence against women in the European Union: A multilevel analysis. J. Epidemiol. Community Health.

[15] Graz C., Etschel E., Schoech H., Soyka M. (2009). Criminal behaviour and violent crimes in former inpatients with affective disorder. J. Affect. Disord..

[16] Peek-Asa C., Zwerling C., Young T., Stromquist A.M., Burmeister L.F., Merchant J.A. (2005). A population based study of reporting patterns and characteristics of men who abuse their female partners. Inj. Prev..

[17] Rode D., Rode M., Januszek M. (2015). Psychosocial characteristics of men and women as perpetrators of domestic violence. Pol. Psychol. Bull..

[18] Shorey R.C., Febres J., Brasfield H., Stuart G.L. (2012). The Prevalence of Mental Health Problems in Men Arrested for Domestic Violence. J. Fam. Violence.

[19] Romans S.E., Poore M.R., Martin J.L. (2000). The perpetrators of domestic violence. Med. J. Aust..

[20] Stuart G.L., Ph D., Meehan J.C., Moore T.M., Morean M., Sc B. (2006). Examining a Conceptual Framework of Intimate Partner Violence in Men and Women Arrested for Domestic Violence. J. Stud. Alcohol.

[21] Swan S.C., Gambone L.J., Caldwell J.E., Sullivan T.P., Snow D.L. (2008). A review of research on women’s use of violence with male intimate partners. Violence Vict..

[22] Swan S.C., Snow D.L. (2002). A Typology of Women’s Use of Violence in Intimate Relationships. Violence Women.

[23] Muftić L.R., Bouffard J.A., Bouffard L.A. (2007). An Exploratory Study of Women Arrested for Intimate Partner Violence: Violent Women or Violent Resistance?. J. Interpers. Violence.

[24] Hester M. (2012). Portrayal of Women as Intimate Partner Domestic Violence Perpetrators. Violence Women.

[25] Falcão De Oliveira S., Ribeiro De Lima Cardoso K., Possante De Almeida C.A., Cardoso L.R., Gutfilen B. (2014). Violence against women: Profile of the aggressors and victims and characterization of the injuries. A forensic study. J. Forensic Leg. Med..

[26] Aaltonen M., Kivivuori J., Martikainen P., Salmi V. (2012). Socio-Economic Status and Criminality as Predictors of Male Violence: Does Victim’s Gender or Place of Occurrence Matter?. Br. J. Criminol..

[27] Antoniou E., Iatrakis G. (2019). Domestic Violence During Pregnancy in Greece. Int. J. Environ. Res. Public Health.

[28] Hing N., O’Mullan C., Mainey L., Nuske E., Breen H., Taylor A. (2021). Impacts of Male Intimate Partner Violence on Women: A Life Course Perspective. Int. J. Environ. Res. Public Health.

[29] Abolarin J., McLafferty L., Carmichael H., Velopulos C.G. (2019). Family Can Hurt You the Most: Examining Perpetrators in Multiple Casualty Events. J. Surg. Res..

[30] Piquero A.R., Theobald D., Farrington D.P. (2014). The overlap between offending trajectories, criminal violence, and intimate partner violence. Int. J. Offender Ther. Comp. Criminol..

[31] Baúto R.V., Carreiro A.F., Pereira M., Guarda R., Almeida I. (2021). Personality and Aggressive Behavior: The Relation between the Five-Factor and Aggression Models in a Domestic Violence Suspects Sample. Med. Sci. Forum.

[32] Kraanen F.L., Scholing A., Emmelkamp P.M.G. (2012). Substance use disorders in forensic psychiatry: Differences among different types of offenders. Int. J. Offender Ther. Comp. Criminol..

[33] Salari S. (2007). Patterns of intimate partner homicide suicide in later life: Strategies for prevention. Clin. Interv. Aging.

[34] Fridel E.E., Zimmerman G.M. (2019). Putting homicide followed by suicide in context: Do macro-environmental characteristics impact the odds of committing suicide after homicide?. Criminology.

[35] Akiskal H.S., Kilzieh N., Maser J.D., Clayton P.J., Schettler P.J., Traci Shea M., Endicott J., Scheftner W., Hirschfeld R.M.A., Keller M.B. (2006). The distinct temperament profiles of bipolar I, bipolar II and unipolar patients. J. Affect. Disord..

[36] Pétursson H., Gudjónsson G.H. (1981). Psychiatric aspects of homicide. Acta Psychiatr. Scand..

[37] Fazel S., Grann M. (2004). Psychiatric morbidity among homicide offenders: A Swedish population study. Am. J. Psychiatry.

[38] Schanda H., Knecht G., Schreinzer D., Stompe T., Ortwein-Swoboda G., Waldhoer T. (2004). Homicide and major mental disorders: A 25-year study. Acta Psychiatr. Scand..

[39] Eronen M., Hakola P., Tiihonen J. (1996). Factors associated with homicide recidivism in a 13-year sample of homicide offenders in Finland. Psychiatr. Serv..

[40] Fatoye F.O., Fatoye G.K., Oyebanji A.O., Ogunro A.S. (2006). Psychological characteristics as correlates of emotional burden in incarcerated offenders in Nigeria. E. Afr. Med. J..

[41] Yu R., Nevado-Holgado A.J., Molero Y., D’Onofrio B.M., Larsson H., Howard L.M., Fazel S. (2019). Mental disorders and intimate partner violence perpetrated by men towards women: A Swedish population-based longitudinal study. PLoS Med..

